# Supplementary material for: Correlation between Oral Health-Related Quality of Life and Objectively Measured Oral Health in People with Ehlers–Danlos Syndromes
Source: Int J Environ Res Public Health. 2020 Nov 8;17(21):8243. doi: 10.3390/ijerph17218243 (PMC7664661; doi:10.3390/ijerph17218243)
Supplement: Supplementary file 1 [file ijerph-17-08243-s001.zip › ijerph-970260-supplementary/S3 Table Participants.pdf]

**Table S3.** Overview of each Participant with corresponding age, sex, OHIP- and PhOX-Score

| Patient ID | Sex[1] | Age | OHIP Score | PhOX Score |
|---|---|---|---|---|
| 1 | W | 58 | 37 | 79 |
| 2 | W | 77 | 17 | 76 |
| 3 | W | 53 | 10 | 69 |
| 4 | W | 61 | 24 | 66 |
| 5 | W | 70 | 23 | 65 |
| 6 | W | 42 | 20 | 64 |
| 7 | W | 53 | 33 | 67 |
| 8 | W | 58 | 13 | 59 |
| 9 | W | 40 | 10 | 75 |
| 10 | W | 47 | 21 | 70 |
| 11 | W | 45 | 13 | 66 |
| 12 | W | 53 | 29 | 73 |
| 13 | W | 43 | 38 | 81 |
| 14 | W | 59 | 18 | 72 |
| 15 | W | 30 | 17 | 74 |
| 16 | W | 39 | 8 | 83 |
| 17 | W | 59 | 42 | 42 |
| 18 | W | 38 | 13 | 76 |
| 19 | W | 39 | 4 | 76 |
| 20 | W | 37 | 7 | 73 |
| 21 | W | 32 | 17 | 72 |
| 22 | W | 25 | 9 | 88 |
| 23 | W | 22 | 2 | 83 |
| 24 | M | 24 | 6 | 91 |
| 25 | W | 64 | 12 | 57 |
| 26 | M | 46 | 0 | 74 |

| | | | | |
|---|---|---|---|---|
| 27 | W | 82 | 16 | 54 |
| 28 | W | 47 | 14 | 69 |
| 29 | W | 45 | 33 | 64 |
| 30 | W | 20 | 6 | 86 |
| 31 | M | 50 | 3 | 70 |
| 32 | W | 33 | 24 | 87 |
| 33 | W | 22 | 26 | 79 |
| 34 | W | 37 | 33 | 58 |
| 35 | W | 29 | 42 | 80 |
| 36 | W | 36 | 31 | 76 |
| 37 | W | 51 | 28 | 38 |
| 38 | W | 25 | 13 | 71 |
| 39 | W | 19 | 36 | 81 |
| 40 | W | 24 | 13 | 84 |
| 41 | W | 37 | 40 | 79 |
| 42 | W | 33 | 33 | 58 |
| 43 | M | 39 | 35 | 63 |
| 44 | W | 47 | 2 | 55 |
| 45 | W | 20 | 2 | 87 |
| 46 | W | 21 | 7 | 83 |

[1]W-female, M-male
