# Supplementary material for: Correlation between Oral Health-Related Quality of Life and Objectively Measured Oral Health in People with Ehlers–Danlos Syndromes
Source: Int J Environ Res Public Health. 2020 Nov 8;17(21):8243. doi: 10.3390/ijerph17218243 (PMC7664661; doi:10.3390/ijerph17218243)
Supplement: Supplementary file 1 [file ijerph-17-08243-s001.zip › ijerph-970260-supplementary/S2 OHIP-G14.pdf]

Bitte zurücksenden an: seltene.erkrankungen@gmail.com oder per Post: Waldeyerstr. 30, 48149 Münster z. Hd. Dr. med. dent. Marcel Hanisch

Fragebogen zur mundgesundheitsbezogenen Lebensqualität von Menschen mit Seltenen Erkrankungen

- Wie alt sind Sie?

- Welches Geschlecht haben Sie?

- Von welcher Seltenen Erkrankung sind Sie betroffen?

- Wie alt waren Sie bei der Diagnose Ihrer Erkrankung?

- Wie viel Zeit ist zwischen dem erstmaligen Auftreten von Symptomen und der definitiven Diagnose Ihrer Erkrankung vergangen?

- Liegt bei Ihrer Erkrankung eine Beteiligung der Mundhöhle oder des Gesichts vor? (z.B. Nichtanlagen von Zähnen, Formanomalien von Zähnen, Missverhältnis von Ober- zu Unterkiefer, Schmelzfehlbildungen der Zähne, Lippen-Kiefer-Gaumenspalte, etc.)

  Wenn ja, welche?

- Konnte durch ein Symptom in der Mundhöhle oder im Gesicht eine Verdachtsdiagnose bezüglich Ihrer Erkrankung gestellt werden?

Wenn ja, durch welches Symptom?

- Hatten Sie Schwierigkeiten, einen Zahnarzt zu finden, der Sie zahnmedizinisch behandelt?

- Werden oder wurden Sie kieferorthopädisch behandelt (Zahnspangen)?

- Wie viel Anfahrtszeit benötigen Sie im allgemeinen bis zu Ihrem Zahnarzt?

- Wie zufrieden sind Sie mit Ihrem jetzigen Zahnarzt? (sehr zufrieden, zufrieden, weniger zufrieden, unzufrieden)

- Fühlen Sie sich vom deutschen Gesundheitssystem in Bezug auf Ihre zahnmedizinische Versorgung ausreichend unterstützt?

- Kennen Sie eine medizinische Versorgungseinrichtung oder ein Expertenzentrum für Ihre Erkrankung? (auch nicht-zahnmedizinische!)

- Kennen Sie eine Informationsquelle zu Ihrer Erkrankung (z.B. Orphanet/ se-atlas/ ROMSE)?

Wenn ja, welche?

Bitte prüfen Sie für jede der folgenden Aussagen, wie sehr die beschriebene Situation für Sie persönlich zutrifft. Kreuzen Sie bitte jeweils die entsprechende Aussage mit einem „x" an.

**Hatten Sie <u>im vergangenen Monat</u> aufgrund von Problemen mit Ihren Zähnen, im Mundbereich oder mit Ihrem Zahnersatz …**	**sehr oft**	**oft**	**ab und zu**	**kau m**	**nie**
….Schwierigkeiten bestimmte Worte auszusprechen?	☐	☐	☐	☐	☐
….das Gefühl, Ihr Geschmackssinn war beeinträchtigt?	☐	☐	☐	☐	☐
….den Eindruck, dass Ihr Leben ganz allgemein weniger zufriedenstellend war?	☐	☐	☐	☐	☐
….Schwierigkeiten zu entspannen?	☐	☐	☐	☐	☐
**Ist es <u>im vergangenen Monat</u> aufgrund von Problemen mit Ihren Zähnen, im Mundbereich oder mit Ihrem Zahnersatz vorgekommen, …**	**sehr oft**	**oft**	**ab und zu**	**kau m**	**nie**
….dass Sie sich angespannt gefühlt haben?	☐	☐	☐	☐	☐
….dass Sie Ihre Mahlzeiten unterbrechen mussten?	☐	☐	☐	☐	☐
….dass es Ihnen unangenehm war, bestimmte Nahrungsmittel zu essen?	☐	☐	☐	☐	☐
….dass Sie anderen Menschen gegenüber eher reizbar gewesen sind?	☐	☐	☐	☐	☐
….dass es Ihnen schwergefallen ist, Ihren alltäglichen Beschäftigungen nachzugehen?	☐	☐	☐	☐	☐
….dass Sie vollkommen unfähig waren, etwas zu tun?	☐	☐	☐	☐	☐
….dass Sie sich ein wenig verlegen gefühlt haben?	☐	☐	☐	☐	☐
….dass Ihre Ernährung unbefriedigend gewesen ist?	☐	☐	☐	☐	☐
**Hatten Sie <u>im vergangenen Monat</u> …**	**sehr oft**	**oft**	**ab und zu**	**kau m**	**nie**
….Schmerzen im Mundbereich?	☐	☐	☐	☐	☐
….ein Gefühl der Unsicherheit in Zusammenhang mit Ihren Zähnen, Ihrem Mund oder Ihrem Zahnersatz?	☐	☐	☐	☐	☐
