# Supplementary material for: Correlation between Oral Health-Related Quality of Life and Objectively Measured Oral Health in People with Ehlers–Danlos Syndromes
Source: Int J Environ Res Public Health. 2020 Nov 8;17(21):8243. doi: 10.3390/ijerph17218243 (PMC7664661; doi:10.3390/ijerph17218243)
Supplement: Supplementary file 1 [file ijerph-17-08243-s001.zip › ijerph-970260-supplementary/S1 PhOX_Erhebung_v1_1.pdf]

Datum:____________ Patienten ID: _________________

A Selbstangaben

„Wie oft hatten Sie in den letzten 30 Tagen im Mund-, Kiefer oder Gesichtsbereich ...?“

Schmerzen:	☐ nie<br>☐ selten (ca. 1x im Monat)<br>☐ ab und zu (mehrfach pro Monat)<br>☐ oft (mehrfach pro Woche)<br>☐ sehr oft (täglich)
Missempfindungen:<br>(Brennen, Taubheitsgefühl, Hypersensitivitäten, Geschmacksveränderungen etc.)	☐ nie<br>☐ selten (ca. 1x im Monat)<br>☐ ab und zu (mehrfach pro Monat)<br>☐ oft (mehrfach pro Woche)<br>☐ sehr oft (täglich)

B Extraoraler Befund

Druckdolenz:<br>(M. temporalis, M. masseter, Kiefergelenk, Gl. parotis, Gl. submandibularis)	☐ nein	☐ ja → *Intensität und Bekanntheit*
	Intensität	☐ leicht<br>☐ mäßig<br>☐ stark
	Bekanntheit:	☐ unbekannt<br>☐ bekannt

C Intraoraler Befund

18	17	16	15	14	13	12	11	21	22	23	24	25	26	27	28
X															X
X															X
48	47	46	45	44	43	42	41	31	32	33	34	35	36	37	38

Legende:
- **S** = gesund, kein Behandlungsbedarf
- **F** = gefüllte, überkronte Zähne (auch implantatgetragene Kronen)
- **D** = kariöse, defekte Zähne
- **R** = ersetzte Zähne (Brückenglied/ Prothesenzahn)
- **M** = fehlende Zähne (Lücke)
- **)(** = Lückenschluss

Anzahl Zähne gesund: **S**	____ [0-28]
Anzahl Zähne gefüllt: **F**	____ [0-28]
Anzahl Zähne defekt: **D**	____ [0-28]
Anzahl Zähne gesamt: **S** + **F** + **D** + **)(**	____ [0-28]

Anzahl Zähne Sondierungstiefe 3,5 - 5,5 mm:	____ [0-6]	**Info:** Ramfjord Zähne: 16, 21, 24, 36, 41, 44
Anzahl Zähne Sondierungstiefe > 5,5 mm:	____ [0-6]	
Blutung bei Sondierung:	☐ nein ☐ ja	
Anzahl perkussionsempfindliche Zähne:	____ [0-28]	

© 2015 UKE Reißmann et al.

Anzahl der Stützzonen:	____ [0-4]	**Info:** mit Zahnersatz
Overjet (sagittaler Überbiss):<br>Overbite (vertikaler Überbiss):<br>Größte Kontaktpunktabweichung:<br>Seitlicher Kreuzbiss:	____ mm<br>____ mm<br>____ mm<br>☐ nein ☐ ja	**Info:** mit Zahnersatz
Mundöffnungsbewegung:<br><br>max. aktive Mundöffnung:<br>max. passive Mundöffnung:	☐ gerade<br>☐ nicht gerade<br>____ mm<br>____ mm	**Info:** mit Zahnersatz
Spalten oder Defekte:	☐ keine<br>☐ operierte Spalte ohne Defekt<br>☐ nur im Weichgewebe<br>☐ knöcherner Defekt ohne MAV<br>☐ knöcherner Defekt mit MAV	
Befeuchtung:<br>(Mundschleimhäute/Lippe/Zunge)	☐ feucht<br>☐ partiell trocken<br>☐ komplett trocken	
Speichelkonsistenz:	☐ flüssig<br>☐ leicht schaumig<br>☐ kein Speichel	
Schwellung:<br>(Mundschleimhäute/Lippe/Zunge)	☐ nein ☐ ja → *Fläche und Intensität*<br>Fläche: ☐ max. ¼ der Oberfläche<br>☐ max. ½ der Oberfläche<br>☐ über ½ der Oberfläche<br>☐ gesamte Oberfläche<br>Intensität: ☐ leicht<br>☐ stark	
Verfärbung:<br>(Mundschleimhäute/Lippe/Zunge)	☐ nein ☐ ja → *Fläche und Intensität*<br>Fläche: ☐ max. ¼ der Oberfläche<br>☐ max. ½ der Oberfläche<br>☐ über ½ der Oberfläche<br>☐ gesamte Oberfläche<br>Intensität: ☐ leicht<br>☐ stark	
Integritätsverlust:<br>(Mundschleimhäute/Lippe/Zunge)	☐ nein ☐ ja → *Fläche und Intensität*<br>Fläche: ☐ max. ¼ der Oberfläche<br>☐ max. ½ der Oberfläche<br>☐ über ½ der Oberfläche<br>☐ gesamte Oberfläche<br>Intensität: ☐ leicht<br>☐ stark	

© 2015 UKE Reißmann et al.
